# Diversity of Metal-Resistant and Tensoactive-Producing Culturable Heterotrophic Bacteria Isolated from a Copper Mine in Brazilian Amazonia

**DOI:** 10.1038/s41598-020-62780-8

**Published:** 2020-04-10

**Authors:** Vitor Sousa Domingues, Andrea de Souza Monteiro, Aline Daniela Lopes Júlio, Ana Luiza Lemos Queiroz, Vera Lúcia dos Santos

**Affiliations:** 1 Laboratory of Applied Microbiology, Department of Microbiology, Institute of Biological Sciences, Universidade Federal de Minas Gerais. Address: Avenida Presidente Antônio Carlos, 6627 – Pampulha/ICB, Bloco F4, sala 159, C.P. 486, Belo Horizonte, MG 31270-901 Brazil; 2 Laboratory of Applied Microbiology, Universidade CEUMA, UNICEUMA, Address: Rua Josué Moentello, Jardim Renascença, São Luís, MA CEP 65075120 Brazil

**Keywords:** Environmental microbiology, Environmental microbiology, Microbial ecology, Microbial ecology

## Abstract

Bacterial extracellular polymeric substances (EPSs) present diverse properties of biotechnological interest, such as surface modification, metal adsorption and hydrophobic substances solubilization through surface tension reduction. Thus, there is a growing demand for new producing strains and structurally variable biomolecules with different properties. One approach for scanning this biodiversity consists of exploring environments under selective pressures. The aim of this study was to evaluate the composition of culturable heterotrophic bacterial communities from five different sites from a copper mine in the Amazon biome by an enrichment technique to obtain metal resistant bacteria (lead, arsenic, cadmium, copper and zinc) capable of producing EPSs. The bacterial densities at the sites varied from 2.42 × 10^3^ to 1.34 × 10^8^ NMP mL^−1^ and the 77 bacterial isolates obtained were classified in four divisions, β-Proteobacteria (16.88%), γ-Proteobacteria (7.29%), Firmicutes (61%) and Actinobacteria (12.98%). *Bacillus*, *Alcaligenes*, and *Lysinibacillus* were the most dominant among the 16 observed genera, but the relative frequency of each varied according to the sample and the metal used in the enrichment culture. 58% of the bacterial strains (45) could produce EPSs. From these, 33 strains showed emulsifying activity (E_24_), and 9 of them reached values higher than 49%. Only *Actinomyces viscosus* E3.Pb5 and *Bacillus subtilis* group E3.As2 reduced the medium surface tension to values lower than 35 mN m^−1^. It was possible to confirm the high presence of bacteria capable of producing EPSs with tensoactive properties in Amazon copper mines and the evolutionary pressure exerted by the heavy metals during enrichment. These molecules can be tested as an alternative for use in processes that involve the removal of metals, such as the bioremediation of contaminated environments.

## Introduction

Amazon forest harbors the greatest biodiversity on Earth and is the world’s largest remaining tropical forest^1^. However, the region undergoes an increasing mining activity as a consequence of its vast potential for mineral assets and the high demand for these resources^2^.

Metal mining has been crucial for human society since the Iron and Bronze Ages^3^. But nowadays, mining faces challenges to efficiently recover metals from the low-grading ores, treat all the waste generated, and remediate the affected areas. The activity reshapes the surrounding environment and has often been associated with air, land, and water pollution^4^.

Microbial communities are also affected by ecosystem transformations engendered by mining activity, as the autochthonous microbiota is molded by abiotic and biotic components^5,6^. In artificial environments, such as in mining tailing dams, microorganisms undergo harsh conditions, as pH variations and the effect of chemical compounds discharged during the process^7^.

To deal with the pollution problem and environmental stressors, adapted microbes present diverse metabolic and physiological strategies. One mechanism is the production of extracellular polymeric substances (EPSs), which potentially increase microbial resistance to high heavy metal concentrations^8^ through ion adsorption onto the molecule functional groups and the prevention of metal penetration into the cytoplasm^9,10^. EPSs can play different parts in the microbial lifecycle and the molecules are predominantly composed of polysaccharides but may also include proteins, lipids, nucleic acids and humic substances^11^. Some exopolymers can also be amphiphilic, showing the ability to accumulate in the interfaces, lowering the interfacial tension and forming micelles, which promote the solubilization of hydrophobic substances in an aqueous phase. Therefore, they can modify surfaces mediating the adhesion and de-adhesion interactions between the microbial cells and interfaces^11,12^. Considering these properties, EPSs have the potential to be employed as heavy metal removers and hydrophobic compound emulsifiers in remediation procedures, depending on their functional groups and on their tensoactives properties, whose effectivity can be measured by their surfactant (mN m^−1^) and emulsifying activities (E_24_ index). Microbial EPSs can also be used as flocculants or frothers in flotation processes^13,14^. The biotechnological applications of these biomolecules are especially noteworthy due to their typical biodegradability and low toxicity^15^.

EPSs are produced by a variety of bacterial species which have been isolated from a wide range of environments, including contaminated soil and water^16^. Nevertheless, there are only few reports of bacterial strains producing these biomolecules isolated from mining sites^17,18^. Therefore, studying mining affected environments would allow us to assess their microbial biodiversity transformations and explore the organisms’ survival strategies as biotechnological applications. The remediation of mining contaminated areas is a huge concern worldwide and remediating technologies based on microorganisms appear as a promising alternative for achieving treatment goals^4^.

Within this context, the aim of this study was to compare the bacterial diversity of five different sites in a copper mine located in the Amazon biome and to reveal differences in the communities regarding the density of cultivable heterotrophic metal-resistant and EPS producing bacteria. This characterization is a step towards the discovery of substances with possible applications in biotechnological and bioremediation processes and an attempt to overcome the limitations that hinder the use of these molecules on a larger scale.

## Results

### Densities of the total heterotrophic bacteria (THB) and the heavy metal-resistant bacteria in the samples

**Figure 1:**
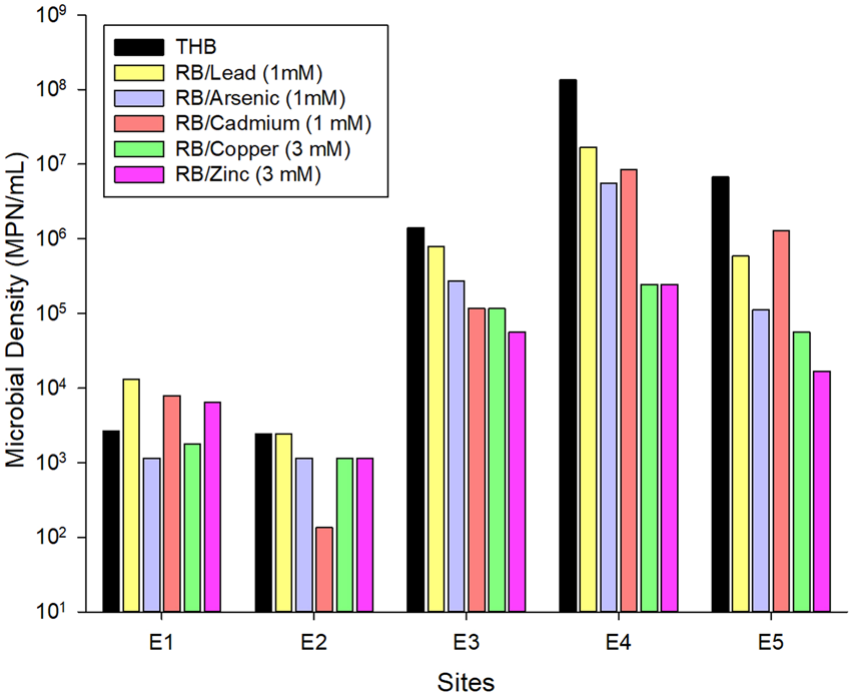
Total and metal-resistant heterotrophic bacterial densities obtained through the Most probable number (MPN) technique from each of the five samples collected on sites E1 (process water), E2 (floater surfaces), E3 (discharging point), E4 (attenuated tailing pond) and E5 (soil). Density of total heterotrophic bacteria (TBH), density of bacteria resistant to lead (RB/Lead), arsenic (RB/Arsenic), cadmium (RB/Cadmium), copper (RB/Copper) or Zinc (RB/Zinc).

Total heterotrophic bacteria **(**THB) densities varied according to the mining site (Fig. 1). In E1 (process water) and E2 (floater surfaces), values reached three orders of magnitude, while in the other samples, the densities surpassed six orders of magnitude, reaching a maximum of 1.34.10^8^ MPN mL^−1^ in E4 (the edge of the tailing pond where the waste particles are sedimented). Despite the higher densities, bacterial communities from E3 (the discharging point where the ore processing wastes are thrown in the tailing pond), E4, and E5 (the soil close to the edge of the tailing pond affected by the discharged waste) showed more sensitivity to heavy metals in MPN assays and when cultured in media with metals (Fig. 1). E3 and E4 samples were affected by all metals. In E3 zinc reduced densities from six to four orders of magnitude. In E4, arsenic and cadmium reduced values from eight to six orders of magnitude, and copper and zinc to five orders. In E5, the presence of cadmium did not cause any expressive change in density, but arsenic and lead induced a decrease from six to five orders of magnitude, and copper and zinc to four orders. On the other hand, in samples E1 and E2 metal negative effects were not observed, except for cadmium in sample E2, where bacterial density was reduced from four to three orders of magnitude.

**Figure 2:**
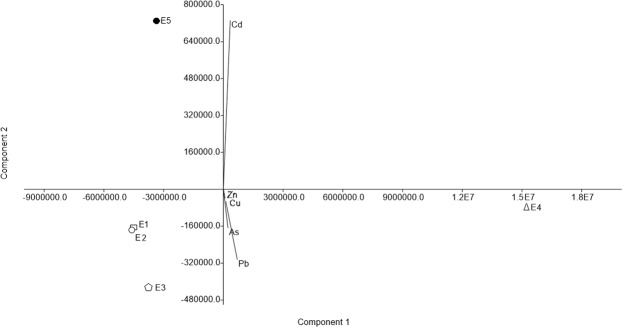
Principal Component Analysis (PCA) of the samples metal concentrations using software PAST 3.04. The axes correspond to the metals and the samples are represented by different symbols.

Principal component analysis (PCA) allowed to assess the metals that most contributed to the variability between samples (Fig. 2). In the analysis, 99.9% of the total variability was explained by the two principal components, almost all by PC1 (99.7%). We observed a group formed by samples E1 and E2, which presented lower density of heavy metal resistant bacteria. The other samples were not groupable because they presented different profiles with a wide cell density variation. Sample E4 presented the highest densities of heavy metal resistant bacteria, while E3 and E5 showed intermediate values, except for the high values with Pb enrichment in sample E3 and Cd enrichment in sample E5.

### Isolation of heavy metal-resistant bacteria through the enrichment technique

**Figure 3:**
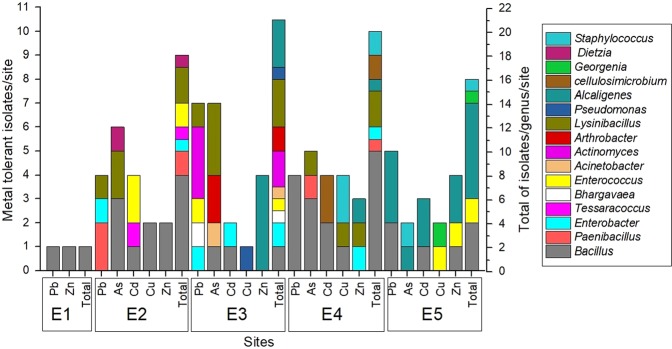
Number of metal-tolerant bacteria isolated from enrichment cultures. The main axis (left) shows the number of isolates tolerant to each metal and from the site specified on the X-axis. The total number of isolates tolerant of all metals at each site is shown on the secondary axis (right). Colour represents the genera.

In the experiment, 77 morphologically distinct bacterial colonies were isolated from the 25 enrichment cultures through the serial dilution plating. Among the bacterial isolates, 21 were obtained from the lead culture, 20 from the arsenic one, 13 from cadmium, 9 from copper, and 14 from zinc. Only two bacterial isolates were obtained from sample E1, 18 from E2, 21 from E3, 20 from E4, and 16 from E5 (Fig. 3).

16S rDNA sequence analysis showed that all isolates were grouped in 23 species from 16 genera from four different phyla: *Bacillus*, *Paenibacillus, Lysinibacillus, Enterococcus*, *Bhargavaeae*, and *Staphylococcus* from Firmicutes phylum (61%); *Tessarococcus, Actinomyces*, *Arthrobacter, Cellulosimicrobium, Georgenia* and *Dietzia* from Actinobacteria phylum (12.98%); *Enterobacter, Acinetobacter* and *Pseudomonas* from the γ-Proteobacteria class from Proteobacteria phylum (7.29%) and *Alcaligenes* from the β-Proteobacteria class from Proteobacteria phylum (16.88%). In E1 sample, only Firmicutes phylum members were observed. E2 sample was also dominated by Firmicutes isolates (83.3%) followed by Actinobacteria (11.1%) and γ-Proteobacteria (5.6%), whereas no β-Proteobacteria was detected. 38% of E3 sample isolates consisted of Firmicutes, 23.8% were Actinobacteria and 19% were γ and β-Proteobacteria. In E4 sample, 80% of bacterial isolates were also Firmicutes and 10% were Actinobacteria, while γ-Proteobacteria and β-Proteobacteria class members corresponded to 5% each. In E5 samples, 50% of the isolates otherwise consisted of β-Proteobacteria while Firmicutes and Actinobacteria members represented 43.7% and 6.3%, respectively, and there was no γ-Proteobacteria isolate detected.

**Figure 4:**
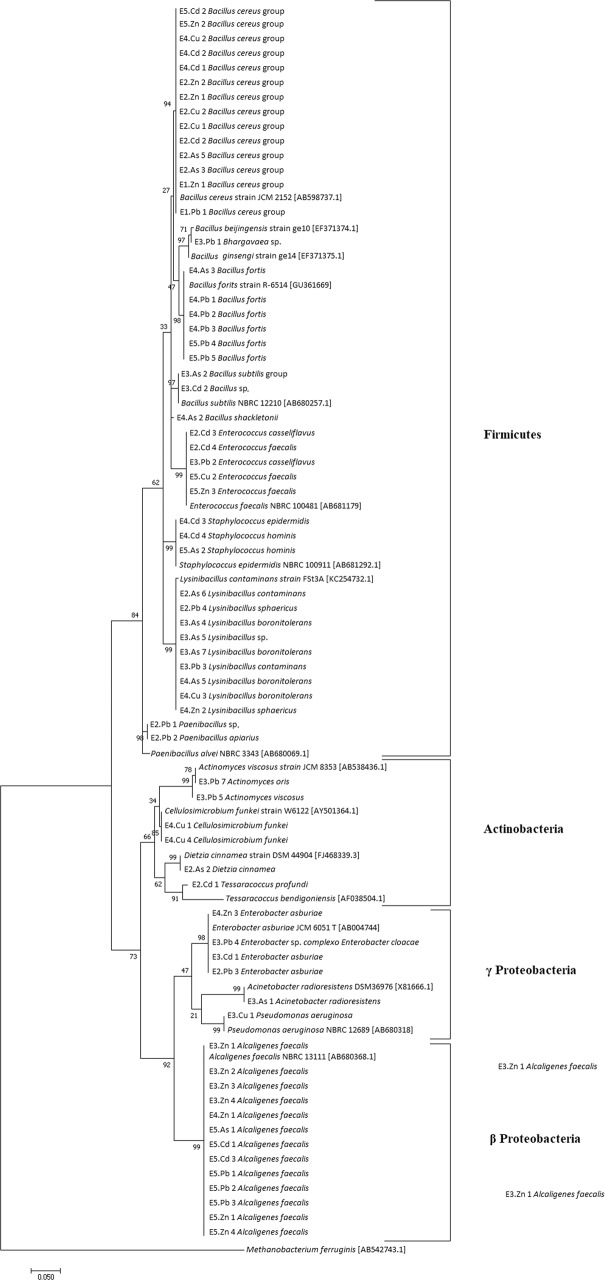
Maximum-likelihood tree indicating phylogenetic relationships of the bacterial isolates from the copper mine and reference sequences from 16S rRNA gene of all obtained genera downloaded from the GenBank database. The four clusters formed for isolates from Firmicutes phylum, Actinobacteria phylum and γ-Proteobacteria and β-Proteobacteria class from Proteobacteria phylum are delimited by braces. The numbers in each grouping were determined by 1000 bootstraps and are show next to the branches.

In general, most of the clusters formed during group phylogenetic analyses supported our molecular taxonomic identification (Fig. 4). We observed the formation of four distinct clades comprising the members from Firmicutes and Actinobacteria phylum and γ-Proteobacteria and β-Proteobacteria classes from Proteobacteria phylum and sub-groups from each genus. Only the Firmicutes clade was composed of bacterial isolates from all five samples. Actinobacteria and γ-Proteobacteria clades were composed of bacterial isolates from the E2, E3 and E4 samples, and the β-Proteobacteria clade by bacterial isolates from the E3, E4 and E5 samples. There was no clear separation of isolates from the same sample in distinct branches of the trees. No separation could also be verified according to the metal used in the enrichment cultures, although no bacterial isolate from Zn enrichment cultures was observed in the Actinobacteria clade or from Cu enrichment cultures in β-Proteobacteria clade (Fig. 4).

Cell density of each bacterial genus was determined in every enrichment culture (Supplementary Table S1). No growth was verified when E1 sample was cultured with 3 mmol L^−1^ of arsenic or cadmium nor with 15 mmol L^−1^ of copper. All other bacterial culture densities varied from 6.1 × 10^2^ UFC mL^−1^, in the culture of E2 added with 3 mmol L^−1^ copper, until 1.45 × 10^8^ UFC mL^−1^, in the culture of E3 added with 15 mmol L^−1^ zinc.

The relative frequency varied according to the genera and the metal used in enrichment culture. In E1 sample, only *Bacillus* isolates were observed in the enrichment cultures with lead and zinc. In E2 sample, *Bacillus* predominated in the enrichments using copper, zinc and arsenic and *Tessaracoccus* predominated in the enrichment using cadmium. On the other hand, in the enrichment using up to 3 mmol L^−1^ of lead, no single genus was dominant, In this culture, *Paenibacillus* and *Enterobacter* genera co-dominated, representing 50.36% and 49.64% of the microbial density, respectively. In E3, each enrichment culture showed one different dominant genus, while in E4, *Bacillus* was the dominant genus in cultures using arsenic and lead. On the other hand, in this culture *Staphylococcus, Cellulosimicrobium and Alcaligenes* predominated in the enrichments using cadmium, copper and zinc, respectively. Finally, a more homogeneous pattern was observed in E5, in which four bacterial cultures, using lead, arsenic, cadmium, and zinc, showed a dominance of the genus *Alcaligenes* always greater than 97%.

Considering the bacterial community structures in metal enriched samples cultures, some bacterial species were found in specific samples, while others were observed in more than one culture. There was no shared species among all the samples, but *Bacillus cereus* group was found in samples E2, E4 and E5, *A. faecalis* in samples E3, E4 and E5 and *Enterobacter asburiae* in samples E2, E3 and E4. In addition, samples E2 and E3 showed the species *Lysinibacillus contaminans* in common, while samples E3 and E4 shared the species *Lysinibacillus boronitolerans* and samples E4 and E5 the species *Bacillus fortis*.

**Figure 5:**
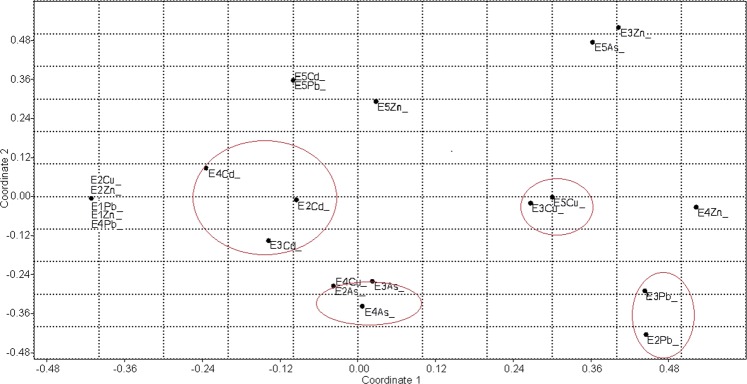
Non-metric multidimensional scaling analysis (NMDS) of the composition of the bacterial community as a function of the metal used in the enrichment using a Jaccard metric in software PAST 3.04.

Through non-metric multidimensional scaling (NMDS) analysis, it was possible to observe the formation of several groups of samples collected in different mining sites as a function of the metal used in the enrichment. A group consisting of two samples enriched with lead (at E2 and E3), another one formed by two samples enriched with copper (at E3 and E5), a group formed by three samples enriched with arsenic (at E2, E3 and E4) and a group formed by three samples enriched with cadmium (at E2, E3 and E4) (Fig. 5).

### Characterization of culture supernatants surface activities of EPS producing bacterial isolates

Screening for EPS-producing bacteria resulted in 34 positive bacterial isolates in the Congo Red based test, and 39 in calcofluor medium test (data not shown).

**Figure 6:**
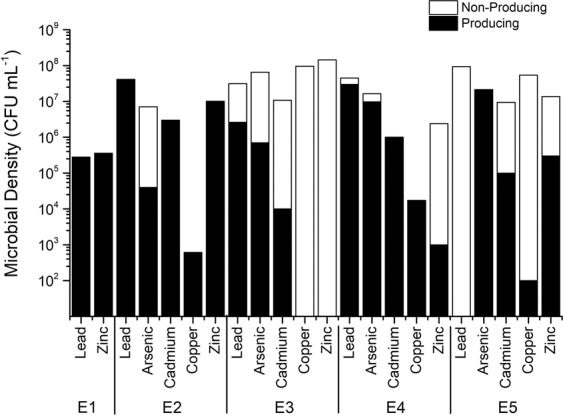
Density of the microbial exopolysaccharide-producing bacteria in the enrichment cultures.

In general E1, E2 and E4 samples showed the highest percentage of EPS producing bacteria (Fig. 6). The values corresponded to 100%, 88.61% and 62.2% in the E1, E2 and E4 samples, respectively, and only 0.95% in E3 and 11.33% in E5.

**Figure 7:**
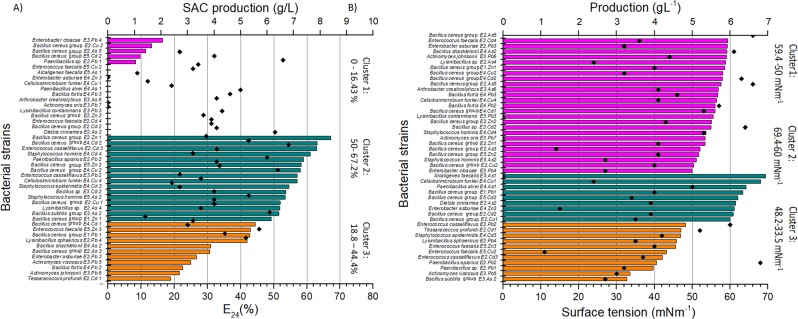
Grouping of bacterial strains in clusters considering the E_24_ (%) (**A**) and surface tension (mN m^−1^) (**B**) values using the k-means algorithm. The clusters are differentiated by colour: pink (cluster 1), green (Cluster 2) and orange (Cluster 3), and the ranges of activities are indicated above the bars. The diamonds show SAC production (g L^-1^) of bacterial strains.

Among the studied microorganisms, only in genera *Bhargavaea, Acinetobacter, Pseudomonas* and *Georgenia*, EPS producing isolates were not observed. We also evaluated the 45 bacterial strains showing positive results for at least one of these tests as well as the production of EPS surface-active compounds in glucose added medium. These bacterial isolates were grouped in three clusters by k-means algorithm considering the culture supernatants E_24_ (%) and surface tension (mN m^−1^) values (Fig. 7A,B). In emulsifying activity assays, 16 bacterial isolates presented indexes greater than 50%, being 1 from genus *Lysinibacillus* (E2.As4), 1 *Paenibacillus* (E2.Pb2), 1 *Cellulosimicrobium* (E4.Cu4), 2 *Enterococcus* (E3.Pb2, E2.Cd3), 3 *Staphylococcus* (E4.Cd3, E4.Cd4, E5.As2), and 8 *Bacillus* (E2.Cu 1, E2.Zn 1, E3.As2, E4.Cd2, E3.Cd2, E4.Cu2, E1.Zn1, E5.Zn2). Another group with 11 bacterial isolates showed activity between 18.8% and 44.4%. The last 18 bacterial isolates presented values below 16.43% and 13 of these did not present any emulsifying activity (Fig. 7A). In the surface tension measurements, only 11 isolates could reduce the media surface tension to values below 48.2 mN m^−1^, highlighting *Actinomyces viscosus* E3.Pb5 and *Bacillus* sp. E3.As2, which reached values lower than 34 mN m^−1^. Exopolysaccharide production varied between 1.1 and 6.8 g.L^−1^, and 50% of the bacterial isolates showed values greater than 4 g L^−1^ (Fig. 7B).

## Discussion

THB density of samples collected from the Sossego Mine ranged from three orders of magnitude in E1 and E2 to eight orders of magnitude in E4. While bacterial densities in E3, E4 and E5 varied more remarkably under heavy metal pressure, E1 and E2 populations showed less sensitivity to the metals. The highest decreases in density in E3, E4 and E5 were mainly in response to copper and zinc.

The lower bacterial densities found at E1 may be the result of an oligotrophic environment in this site, with low levels of P (0.02 mg L^−1^) and ammoniacal N (0.2 mg L^−1^), organic matter (DBO < 3 mg L^−1^), and other nutrients, and at E2 due to an adverse environment with an excess of minerals and chemical substances, such as frothers and collectors used for recovering chalcopyrite in the flotation process. In general, oligotrophic freshwater environments in Brazil show P and N concentrations below 0.03 and 0.6 mg L^−1^, respectively^19^. Phosphorus level in E1 sample was in the range found in other ecosystems in which bacteria was phosphorus-limited (0.003 to 0.107 mg L^−1^)^20,21^. In E3, E4, and E5 samples, the proximity to the soil and vegetation allows a greater access to nutrients, explaining the higher THB values and the highest sensitivity to heavy metals added to the culture.

It has been suggested that long-term exposition to metals leads to the adaptation of part of the microbial community that then survives in the metal-polluted site. But such effect on the density of culturable bacteria is uncertain due to the contrasting results that has been found. Ansari and Malik^22^ also applied the MPN technique to analyze bacterial communities from soils contaminated with heavy metals in an industrial site over various seasons. Densities, reaching six orders of magnitude, decreased between 74.29% and 45.20%, in the presence of cadmium at 0.89 mmol L^-1^ during some seasons. Hiroki^23^ also found a negative correlation between copper level and the bacterial density, whereas Smit *et al*.^24^, Dell’Amico *et al*.^25^ and Turpeinen *et al*.^26^ found that the HB density was not affected by metal level. Regarding microbial diversity, many studies found similar indexes on soils with different metal concentrations^27,28^, whereas others indicated that both diversity and structure of communities changed following metal contamination^24,26,29^.

Phylum Firmicutes was the only one found in all samples and in most of them, it prevailed, except for E5 sample, in which phylum Proteobacteria predominated due to a high number of isolates from *Alcaligenes faecalis* species. Some studies reported the isolation of bacterial strains belonging to Phylum Firmicutes in environments contaminated with multiple heavy metals^30^ although these isolates are often not the prevailing ones. In the studies conducted by Gillan *et al*.^31^ in marine ecosystems and by Vishnivetskaya *et al*.^32^ in streams, the most predominant phylum in environments contaminated with heavy metals was Proteobacteria. Pepper *et al*.^33^ reported that the most dominant groups in almost all collected samples from an Arizona copper mine belonged to Proteobacteria and Actinobacteria phyla. There were no species in common among all the samples, but due to similarities in the overall composition of groups and considering that distinct clades were formed only as a function of the phyla, no specific clusters were obtained according to each sample or the heavy metal used in enrichment. However, it was possible to support the formation of sample groups of different sites submitted to enrichment with the same metal according to the NMDS. This demonstrates the presence of similar bacteria in the collected original samples and confirms the selection pressure exerted by each metal. Bacterial species or genera relative frequency varied according to the site and the metal used in the enrichment culture. This variation pattern has previously been related^34^.

The genus *Bacillus* dominated seven enrichment cultures and usually prevails in studies performed in heavy metal-contaminated areas, also including those based on enrichment techniques to select metal-resistant microorganisms. As an example, the study conducted by Lenart and Wolny-Koladka^35^ using samples of soil microbiota and phylloplanes at the roadside, containing 0.5 to 1.6 mmol L^−1^ of cadmium, 138.2 to 900.7 mmol L^−1^ of zinc, and 6.1 to 442.1 mmol L^−1^ of lead, showed that 31.51% of the bacterial population belonged to the genus *Bacillus*. In another study, three of four species selected from enrichment, using soil contaminated with heavy metals as the inoculum and medium containing 200 mg L^−1^ of lead and 50 mg L^−1^ of cadmium or copper, were found to belong to this genus^36^. On the other hand, the ability to tolerate heavy metals of the species *Georgenia thermotolerans* and *Dietzia cinnamea*, and the genus *Cellulosimicrobium* and *Tessaracoccus* is poorly known. The first isolates of *G. thermotolerans* were obtained from forest soils in Japan^37^ and from the contaminated soil of an iron mine in India, but resistance to metals was not analyzed^38^. *D. cinnamea* strains capable of degrading hydrocarbon were isolated from contaminated soils in an Atlantic Forest reserve in Brazil^39^ and a strain of *Dietzia* sp was obtained from steel mill wastes containing chromium also in Brazil, showing resistance of up to 2 mmol L^−1^ of copper and lead^40^. Isolates of *Cellulosimicrobium* sp. have also been obtained from soils contaminated with chromium and from tannery effluents contaminated with metal^41,42^. For the genus *Tessaracoccus*, a report of the isolation from metalworking fluid of the species *T. lubricantis* was found^43^.

EPS production was verified by 58% of the bacterial isolates. The variation of EPS producing bacteria in each sample may be related to the pressure exerted by the heavy metals during enrichment and dominant species in each one. In the samples E1, E2 and E4, which presented the highest percentages of EPS producers, species of the genus *Bacillus* predominated and of the 20 isolates evaluated, 15 produced EPS. On the other hand, the samples E3 and E5, characterized by low EPS production, were dominated by *A. faecalis*. The results also showed that 12 of the 13 isolates of this species did not produce EPSs. Metabolic pathways for EPS production are widely distributed among bacterial species, however, the production of these biomolecules varies according to the bacterial strain, explaining why not every *A. faecalis* strain produces EPS. All EPS producing bacterial isolates were characterized as surface active compound producers. Seventy four percent of *Bacillus* genus isolates were able to produce EPSs and 24% of non-producing isolates belonged to the species *B. fortis*. Several species of *Bacillus*, especially from *cereus* and *subtilis* groups, are known for their metal ions resistance. Bacterial strains resistant and able to adsorb arsenic, lead, copper, cadmium, and zinc have already been described, usually isolated from heavy metal contaminated areas^44,45^.

The bacteria isolates were grouped in three clusters considering E_24_ (%) and surface tension (mN m^−1^) values. According to Desai and Banat^46^, microorganisms with good surfactant production decrease media surface tension to values near 35 mN m^−1^. The screening methodology aimed to detect the production of EPS, which are polymeric substances. Thus, greater supernatant emulsifying activity was expected as bioemulsifiers are mostly higher molecular mass substances^10^. From the 16 species presenting better emulsifying activity, 8 belonged to the genus *Bacillus*, which also showed high surfactant activity. These indexes reached 67.21% (E_24_) for *Bacillus* sp. E2.Zn1 and a decrease in surface tension for up to 32.75 mN m^−1^ for *Bacillus* sp. E3.As2. The other good emulsifiers belong to the genera *Staphylococcus* (3), *Enterococcus* (2), *Paenibacillus* (1), *Cellulosimicrobium* (1), and *Lysinibacillus* (1). Isolates of genera *Enterococcus* (4), *Paenibacillus* (2), *Staphylococcus* (1), and *Lysinibacillus* (1) were also observed among the group of isolates with better surfactant activity. Other studies have verified the production of surface-active compounds by heavy metal- or hydrocarbon-resistant bacteria of these genera^47–50^.

In conclusion, the choice of the sample collection points provided evidence of the bacteria composing the different sites of the mining environment located in the rich and underexplored Amazon biome. These bacteria could present resistance mechanisms for surviving under many stress conditions. Bacteria resistant to lead, arsenic, cadmium, copper, and zinc were isolated from almost all collection points through the enrichment technique. 58% from the total bacterial strains were able to produce surface-active compounds, suggesting that the production of these molecules may be related to the evolutionary pressure exerted by the heavy metals. Two bacterial isolates were able to reduce culture supernatant surface tension to values below 35 mN m^−1^, and 12 bacterial isolates showed toluene emulsifying indexes higher than 50%. The greatest emulsifying substance production compared to the surfactants may be related to the selection methods, which favor polymeric molecules, which usually presented higher emulsifying indexes. Therefore, in this study, we characterized surface active EPSs producing bacteria capable of growing under high concentrations of heavy metals. The characteristics identified in the producing bacterial isolates contribute to their resilience and make them a practical tool in bioremediation strategies. The study of the structure of these molecules and the optimization of their production may also boost the use of natural surface-active compounds in bioremediation processes beyond others biotechnological applications.

## Material and methods

### Site description, sampling and bacterial isolation

Different samples were collected from a copper mine located in the city of Canaã dos Carajás, in the state of Pará, Brazil, (06°23′14.9″S–50°03′19.5″W, 238 meters above sea level). In 2018, Sossego Mine produced around 92 thousand tons of copper concentrate^51^. In the mine, copper is obtained from chalcopyrite ores (CuFeS_2_) and the raw material is crushed before being processed in a ball mill. After milling, the mineral is recovered in serial floaters using propylene glycol and methyl isobutyl as frothers and amyl xanthate and xanthate ester as collectors. In the sequence, the material passes through a dewatering process, retaining a 30% copper concentrate. The waste generated during floatation, dewatering, thickening, and filtration procedures is discharged in the tailing dam. After self-depuration, water is pumped from the opposite edge of the tailing dam for being reused in the industrial processes.

The samples were collected in five different sites (Supplementary Fig. 1) and consisted of three 150 mL subsamples mixed in sterile plastic bottles, which were stored on ice for 48 h until being processed in a laboratory. The five collecting points were: process water (E1); floater surfaces, (E2); the discharging point where the ore processing wastes are thrown in the tailing pond (E3); the edge of the tailing pond where the waste particles are sedimented (E4); the soil close to the edge of the tailing pond affected by the discharged waste (E5). The water of the tailing pond contained low concentrations of Cu (0.006 mg L^−1^), Ni (<0.001 mg L^−1^), Pb (<0.001 mg L^−1^), As (0.01), Al (0.21 mg L^−1^), Cr (<0.01 mg L^−1^), Cd (<0.01 mg L^−1^), Zn (<0.10 mg L^−1^), Fe^2+^ (0.13 mg L^−1^), and Fe^3+^ (0.1 mg L^−1^), which do not exceed the maximum values established in the Brazilian guidelines for framing water bodies within quality standards^52^. Sulfate concentration was higher, corresponding to 263 mg L^−1^. The site showed a pH of 7.9, a temperature of 27 °C, turbidity of 0.69 NTU, DO of 7.6 mg L^−1^, BOD < 3 mg L^−1^, COD of 8.7 mg L^−1^, P of 0.02 mg L^−1^, K of 33.1 mg L^−1^, ammoniacal nitrogen of 0.2 mg L^−1^, Na of 156 mg L^−1^, sulfite <2 mg L^−1^, and sulfide 0.002 mg L^−1^.

Most probable number (MPN) technique was performed to quantify metal-resistant bacteria and total heterotrophic bacteria (THB) in each sample^53^. To determine THB density, 180 µL of BHI broth (Difco, Detroit, MI, USA) and 20 µL of the diluted samples (10^−1^ to 10^−7^) were added in quadruplicate into the wells of 96-well polystyrene microplates (Sigma, St. Louis, MO). The plates were incubated for seven days at 37 °C and the bacterial respiratory activity, an evidence of microbial growth, was determined by adding to each well 50 μL of 2,3,5-triphenyl chloride tetrazolium (Sigma, St. Louis, MO) at 3.0 g L^−1^. After 24 h incubation at 37 °C, the pink color in the wells, resulting from reagent reduction, was quantified at 485 nm. After determining the number of positive and negative wells for each dilution, the MPN, represented by the value x, was calculated using Microsoft Excel software and the equation described by Briones Jr and Reichardt^54^:

$$\frac{{a}_{1}.{p}_{1}}{1-{e}^{-{a}_{1}.x}}+\frac{{a}_{n}.{p}_{n}}{1-{e}^{-{a}_{n}.x}}={a}_{1}.{n}_{1}+{a}_{n}.{n}_{n}$$

where a is the volume added to each well, p is the number of positive wells, and n is the number of inoculated wells.

To determine the density of the heavy metal resistant HB in the samples, the same procedure was performed, but adding lead, arsenic, and cadmium to the BHI broth at a concentration of 1 mmol L^−1^, or copper and zinc at a concentration of 3 mmol L^−1^. The salts used to achieve the desired metal concentrations were Pb(C_2_H_3_O_2_)_2_·3H_2_O, Na_2_HAsO_4_·7H_2_O, CdCl_2_, CuSO_4_, and ZnSO_4_ (Sigma, St. Louis, MO)_._ Samples were also grouped, considering the most probable number of heavy metal resistant HB, through PCA using PAST software version 3.04.

To develop the enrichment cultures, 10 mL of each sample were inoculated in 100 mL of BHI broth with lead, arsenic, and cadmium added at an initial concentration of 1 mmol L^−1^, or copper and zinc at a concentration of 5 mmol L^−1^. The 25 flasks were incubated for 7 days at 37 °C, and after this period, 10 mL of these cultures were inoculated in a new media containing lead, arsenic, and cadmium at 2 mmol L^−1^, and copper and zinc at 10 mmol L^−1^. A new re-inoculation was performed after 10 days under the same incubation conditions in flasks containing lead, arsenic, and cadmium at 3 mmol L^−1^, and copper and zinc at 15 mmol L^−1^, which were cultivated for another 15 days. Then, culture aliquots were serially diluted in saline solution (0.85% NaCl), and 100 µL of each dilution from 10^0^ to 10^−6^ were plated in BHI agar and incubated at 37 °C for 24 h. After growth, all different morphotypes were characterized and counted to determine microbial density. Different colonies were then cultivated and stored at −80 °C on BHI broth plus 20% glycerol.

### Bacterial identification

Genomic bacterial DNA was extracted using the guanidine thiocyanate method described by Pitcher *et al*.^55^. The concentration and purity of the products were quantified in a Nanodrop^TM^ 1000 spectrophotometer (Thermo Scientific, Wilmington, Delaware USA) at 260 and 280 nm, respectively. To amplify the 16S rDNA region partial sequences, the universal bacterial primers 8 F (5′-AGAGTTTGATCCTGGCTCAG-3′) and 907R (5′-CCGTCAATTCCTTTRAGTTT-3′) were used^56^. The amplification reaction was performed in a final volume of 50 µL, containing 10 µL of Taq IVB (5X) buffer, 2.5 U of Taq DNA polymerase buffered with KCl (Phoneutria, Brazil), 200 µmol L^−1^ of each deoxyribonucleotide (dATP, dCTP, dGTP, and dTTP), 0.3 pmol L^−1^ of each primer, and 40 to 240 ng of DNA. Amplification was performed in a thermocycler (Veriti, Applied Biosystems, Foster City, Califórnia, USA) under the following conditions: initial denaturation at 94 °C for 5 min, followed by 21 cycles with denaturation at 94 °C for 1 min, annealing at 57 °C for 1 min in the first three cycles, then a decrease of 1 °C for each two cycles until a temperature of 49 °C in the last two cycles, and extension at 72 °C for 3 min, ending with a final extension at 72 °C for 10 min^57^. The amplification product concentration and purity were also quantified in a Nanodrop^TM^ 1000 spectrophotometer and the integrity of fragments of roughly 900 bp was analyzed through 10 g L^−1^ agarose gel electrophoresis.

The amplification products were purified with EDTA plus ethanol absolute, and the concentration and purity were evaluated in the Nanodrop^TM^ 1000 spectrophotometer. The sequencing reaction was performed in 96-well plates using a final volume of 10 µL. Twenty ng of the purified amplification product were added to the reaction along with the reaction buffer, BigDye® Terminator v3.1 cycle (Applied Biosystems, Foster City, Califórnia, USA), and the same amplification primers at a concentration of 5 µmol L^−1^. An initial denaturation was performed at 95 °C for 1 min, then 35 cycles were completed with denaturation at 96 °C for 15 s, annealing at 50 °C for 15 s, and extension at 60 °C for 5 min, and a final cooling at 4 °C. The sequencing reaction product was purified using EDTA plus ethanol absolute and the purification product was suspended in Hi-Di^TM^ formamide for analysis in the ABI Prism 3100 sequencer (Applied Biosystems, Foster City, Califórnia, USA).

The nucleotide sequences obtained were trimmed using the Sequencher 4.1.4 software, and, when possible, the reaction products using the different primers were aligned to generate the consensus sequence. Phylogenetic affiliations of these sequences were initially estimated using the GenBank database BLAST tool. When compared, bacterial isolates were considered the same species as the highest score database sequence when the value was greater than 97%^58^. The reference sequences of all found genera (preferably ATCC, CIP, or NCRB) were downloaded from the GenBank database and aligned with the 16S rDNA bacterial isolates’ sequences using the multisequence alignment program ClustalW^59^. Phylogenetic affiliations were additionally inferred from this multiple alignment using the maximum-likelihood method^60^ in the MEGA (Molecular Evolutionary Genetics Analysis 7) software^61^, and topologies of the resulting trees were evaluated using bootstrap analysis^62^ based on 1,000 replicates. *Methanobacterium ferruginis* AB542743.1 16S rDNA gene sequence was used as an out-group. Sequences were deposited in GenBank library and access numbers are provided at Supplementary Table S2.

After bacterial isolates identification, the absolute and relative frequencies of each genera were estimated for the different samples subjected to enrichment. The absolute frequency corresponds to the sum of the count of the same genera colonies in UFC mL^−1^ in each sample. Relative frequency was calculated by dividing the absolute frequency of each genera by the sum of all the absolute frequencies in the sample.

A NMDS analysis was employed to assess the effect of metal enrichments on the general structure of bacterial isolates from the five mining sites, according to the identified genera and using the Jaccard metric in PAST software version 3.04.

### Characterization of exopolysaccharide producing morphotypes

Assays were performed in differential growth media for detecting the production of exopolysaccharides by each morphotype. Each one was inoculated in Congo Red Agar (37 g L^−1^ BHI, 50 g L^−1^ sucrose, 10 g L^−1^ agar, and 0.8 g L^−1^ Congo Red dye), in which producing colonies present dark pigmentation^63^, and in BHI agar supplemented with 0.02 g L^−1^ Calcofluor White M2R (Sigma, St. Louis, MO, USA), in which exopolysaccharide producing colonies fluoresce.

### Characterization of the production of bacterial surface active extracellular polymeric substances

Bacterial strains able to synthesize exopolysaccharides were inoculated at an initial concentration of OD_600nm_ 0.1 in 50 mL of minimum mineral media containing 0.5 g L^−1^ urea, 0.5 g L^−1^ yeast extract, 0.2 g L^−1^ ammonium sulfate, 0.1 g L^−1^ sodium chloride, 0.2 g L^−1^ magnesium sulfate heptahydrate, 5 g L^−1^ potassium phosphate dibasic, and 2 g L^−1^ potassium phosphate monobasic, supplemented with 25 g L^−1^ glucose. Bacterial cultures were incubated for up to 7 days at 37 °C under agitation of 180 rpm. Finally, each culture was centrifuged at 5,000 *g* for 15 min, supernatants were evaluated for surfactant and emulsifying activities, and exopolysaccharides extraction was performed.

The capacities of cell-free culture supernatants to reduce the surface tension of growth media were assessed using a K100C-MK2 tensiometer (Kruss, Hamburg, Germany). Measures were performed at room temperature employing a platinum plate.

Supernatant emulsifying activity was quantified according to Cameron *et al*.^64^. In screw cap test tubes, 1 mL of the supernatant was mixed with 1.5 mL of toluene (Sigma, St. Louis, MO, USA) and vortexed for 2 min. After 24 h, the emulsifying index (E_24_) was determined by dividing the emulsified layer height by the total mixture height, and expressing the result as a percentage. Bacterial isolates were grouped in exclusive clusters using the *k-means* clustering algorithm in PAST software (version 1.90) considering E_24_ (%) and surface tension (mN m^−1^) values.

At last, four volumes of ethanol absolute at 4 °C were added to the supernatants for precipitating EPS from cell-free culture media. The precipitate was removed through centrifugation at 5,000 *g* for 15 min, and after discharging the liquid phase, it was dried at 37 °C for 72 h and weighed to calculate production in g L^−1^.

## Supplementary information

Supplementary info.
