# Supplementary material for: Diversity of Metal-Resistant and Tensoactive-Producing Culturable Heterotrophic Bacteria Isolated from a Copper Mine in Brazilian Amazonia
Source: Sci Rep. 2020 Apr 10;10:6171. doi: 10.1038/s41598-020-62780-8 (PMC7148335; doi:10.1038/s41598-020-62780-8)

**Diversity of Metal-Resistant and Tensoactive-Producing Culturable Heterotrophic Bacteria Isolated from a Copper Mine in Brazilian Amazonia**

Vitor Sousa Domingues ^a^, Andrea de Souza Monteiro ^b^, Aline Daniela Lopes Júlio ^a^, Ana Luiza Lemos Queiroz ^a^, Vera Lúcia dos Santos ^a*^

^a^ Laboratory of Applied Microbiology, Department of Microbiology, Institute of Biological Sciences, Universidade Federal de Minas Gerais. Address: Avenida Presidente Antônio Carlos, 6627 – Pampulha/ICB, Bloco F4, sala 159. Belo Horizonte – MG, C.P. 486, 31270-901, Brazil.

^b^ E-mail: andreasmont@gmail.com. Affiliation: Laboratory of Applied Microbiology, Universidade CEUMA, UNICEUMA, Address: Rua Josué Moentello, Jardim Renascença, São Luís - MA, CEP 65075120, Brazil.

* Corresponding author e-mail: verabio[@gmail.com](mailto:domingues.vtrs@gmail.com). Affiliation: Laboratory of Applied Microbiology, Department of Microbiology, Institute of Biological Science, Universidade Federal de Minas Gerais. Adress: Avenida Presidente Antônio Carlos, 6627 – Pampulha / ICB, Bloco F4, sala 159. Belo Horizonte – MG, C.P. 486, 31270-901, Brazil.

**Supplementary Table S1 online:** Density and relative frequency of each bacterial genera from the enrichment cultures in the five different samples.

| **Bacterial genera** | **Density (CFU.mL^-1^) / Relative frequency (%) of bacterial genera in the samples** | | | | | | | | | | | | | | | | | | | | | | |
| --- | --- | --- | --- | --- | --- | --- | --- | --- | --- | --- | --- | --- | --- | --- | --- | --- | --- | --- | --- | --- | --- | --- | --- |
|  | **E1** | | **E2** | | | | | **E3** | | | | | **E4** | | | | | | **E5** | | | | |
|  | **Pb** | **Zn** | **Pb** | **As** | **Cd** | **Cu** | **Zn** | **Pb** | **As** | **Cd** | **Cu** | **Zn** | **Pb** | **As** | **Cd** | **Cu** | **Zn** | **Pb** | | **As** | **Cd** | **Cu** | **Zn** |
| *Bacillus* | 2.8x10^5 (^100) | 3.6x10^5 (^100) | - | 6.0x10^6^ (85.51) | 2.0x10^4^ (0.66) | 6.1x10^2^ (100) | 1.0x10^7^ (100) | - | 6.0x10^5 (0.92)^ | 1.0x10^4^  ^(0.09)^ | - | - | 4.5x10^7 (100)^ | 1.5x10^7 (94.45)^ | 3.6x10^3 (0.36)^ | 7.0x10^2 (4.04)^ | - | 2.0x10^6 (2.13)^ | | - | 1.0x10^5 (1.06)^ | - | 1.0x10^5 (0.73)^ |
| *Paenibacillus* | - | - | 2.1x10^7^ (50.36) | - | - | - | - | - | - | - | - | - | - | 9.0x10^5 (5.48)^ | - | - | - | - | | - | - | - | - |
| *Enterobacter* | - | - | 2.1x10^7^ (49.64) | - | - | - | - | 4.0x10^5^ (1.27) | - | 1.1x10^7 (99.91)^ | - | - | - | - | - | - | 1.0x10^3^  (0.04) | - | | - | - | - | - |
| *Tessaracoccus* | - | - | - | - | 3.0x10^6^ (99.27) | - | - | - | - | - | - | - | - | - | - | - | - | - | | - | - | - | - |
| *Dietzia* | - | - | - | 1.0x10^4^  (0.14) |  |  |  |  |  |  |  |  |  |  |  |  |  |  | |  |  |  |  |
| *Staphylococcus* | - | - | - | - | - | - | - | - | - | - | - | - | - | - | 1.0x10^6 (99.64)^ |  |  |  | | 1.0x10^4^  (0.05) |  |  |  |
| *Bhargavaea* | - | - | - | - | - | - | - | 2.9x10^7^ (91.72) | - | - | - | - | - | - | - | - | - | - | | - | - | - | - |
| *Enterococcus* | - | - | - | - | 2.0x10^3^  (0.066) | - | - | 1.0x10^5 (0.32)^ | - | - | - | - | - | - | - | - | - | - | | - | - | 1.0x10^3^  (0.002) | 2.0x10^5 (1.46)^ |
| *Actinomyces* | - | - | - | - | - | - | - | 1.8x10^6 (5.73)^ | - | - | - | - | - | - | - | - | - | - | | - | - | - | - |
| *Acinetobacter* | - | - | - | - | - | - | - | - | 6.4x10^7 (98.45)^ | - | - | - | - | - | - | - | - | - | | - | - | - | - |
| *Arthrobacter* | - | - | - | - | - | - | - | - | 2.0x10^5 (0.31)^ | - | - | - | - | - | - | - | - | - | | - | - | - | - |
| *Lysinibacillus* | - | - | 2.0x10^3^  (0.005) | 1.0x10^6^  (14.35) | - | - | - | 3.0x10^5 (0.96)^ | 2.1x10^5 (0.32)^ | - | - | - | - | 1.0x10^4^  ^(0.07)^ | - | 1x10^1^  (0.06) | 1.0x10^5 (4.16)^ | - | | - | - | - | - |
| *Pseudomonas* | - | - | - | - | - | - | - | - | - | - | 9.6x10^7 (100)^ | - | - | - | - | - | - | - | | - | - | - | - |
| *Alcaligenes* | - | - | - | - | - | - | - | - | - | - | - | 1.45x10^8 (100)^ | - | - | - | - | 2.3x10^6 (95.79)^ | 9.2x10^7 (97.87)^ | | 2.1x10^7 (99.95)^ | 9.3x10^6 (98.94)^ | - | 1.3x10^7 (97.81)^ |
| *Cellulosimicrobium* | - | - | - | - | - | - | - | - | - | - | - | - | - | - | - | 1.7x10^4 (95.9)^ | - | - | | - | - | - | - |
| *Georgenia* | - | - | - | - | - | - | - | - | - | - | - | - | - | - | - | - | - | - | | - | - | 5.4x10^7 (100)^ | - |
| **Total** | 2.8x10^5^ | 3.6x10^5^ | 4.2x10^7^ | 7.0x10^6^ | 3.0x10^6^ | 6.1x10^2^ | 1.0x10^7^ | 3.1x10^7^ | 6.5x10^7^ | 1.1x10^7^ | 9.6x10^7^ | 1.45x10^8^ | 4.5x10^7^ | 1.6x10^7^ | 1.0x10^6^ | 1.7x10^4^ | 2.4x10^6^ | 9.4x10^7^ | | 2.1x10^7^ | 9.4x10^6^ | 5.4x10^7^ | 1.4x10^7^ |

**Supplementary Table S2 online:** Molecular identification of the bacteria isolated from the copper mine and Access numbers on GenBank.

| **GenBank accession number** | **Bacterial strains** |
| --- | --- |
| KJ513379.1 | *Alcaligenes faecalis* strain E5.Zn4 |
| KJ513378.1 | *Enterococcus faecalis* strain E5.Zn3 |
| KJ513377.1 | *Bacillus cereus* group strain E5.Zn2 |
| KJ513376.1 | *Alcaligenes faecalis* strain E5.Zn1 |
| KJ513375.1 | *Enterococcus faecalis* strain E5.Cu2 |
| KJ513374.1 | *Georgenia thermotoleran*s strain E5.Cu1 |
| KJ513373.1 | *Alcaligenes faecali*s strain E5.Cd3 |
| KJ513372.1 | *Bacillus* *cereus* group strain E5.Cd2 |
| KJ513371.1 | *Alcaligenes faecali*s strain E5.Cd1 |
| KJ513370.1 | *Staphylococcus hominis* strain E5.As2 |
| KJ513369.1 | *Alcaligenes faecalis* strain E5.As1 |
| KJ513368.1 | *Bacillus fortis* strain E5.Pb5 |
| KJ513367.1 | *Bacillus fortis* strain E5.Pb4 |
| KJ513366.1 | *Alcaligenes faecali*s strain E5.Pb3 |
| KJ513365.1 | *Alcaligenes faecalis* strain E5.Pb2 |
| KJ513364.1 | *Alcaligenes faecali*s strain E5.Pb1 |
| KJ513363.1 | *Enterobacter asburiae* strain E4.Zn3 |
| KJ513362.1 | *Lysinibacillus sphaericu*s strain E4.Zn2 |
| KJ513361.1 | *Alcaligenes faecalis* strain E4.Zn1 |
| KJ513360.1 | *Cellulosimicrobium funkei* strain E4.Cu4 |
| KJ513359.1 | *Lysinibacillus boronitolerans* strain E4.Cu3 |
| KJ513358.1 | *Bacillus cereus* group strain E4.Cu2 |
| KJ513357.1 | *Cellulosimicrobium funke*i strain E4.Cu1 |
| KJ513356.1 | *Staphylococcus homin*is strain E4.Cd4 |
| KJ513355.1 | *Staphylococcus epidermidis* strain E4.Cd3 |
| KJ513354.1 | *Bacillus cereus* group strain E4.Cd2 |
| KJ513353.1 | *Bacillus cereus* group strain E4.Cd1 |
| KJ513352.1 | *Lysinibacillus boronitolerans* strain E4.As5 |
| KJ513351.1 | *Bacillus fortis* strain E4.As4 |
| KJ513350.1 | *Bacillus fortis* strain E4.As3 |
| KJ513349.1 | *Bacillus shackletonii* strain E4.As2 |
| KJ513348.1 | *Paenibacillus alvei* strain E4.As1 |
| KJ513347.1 | *Bacillus fortis* strain E4.Pb4 |
| KJ513346.1 | *Bacillus fortis* strain E4.Pb3 |
| KJ513345.1 | *Bacillus forti*s strain E4.Pb2 |
| KJ513344.1 | *Bacillus fortis* strain E4.Pb1 |
| KJ513343.1 | *Alcaligenes faecalis* strain E3.Zn4 |
| KJ513342.1 | *Alcaligenes faecalis* strain E3.Zn3 |
| KJ513341.1 | *Alcaligenes faecalis* strain E3.Zn2 |
| KJ513340.1 | *Alcaligenes faecali*s strain E3.Zn1 |
| KJ513339.1 | *Pseudomonas aeruginosa* strain E3.Cu1 |
| KJ513338.1 | *Bacillus* sp. strain E3.Cd2 |
| KJ513337.1 | *Enterobacter asburiae* strain E3.Cd1 |
| KJ513336.1 | *Lysinibacillus boronitolerans* strain E3.As7 |
| KJ513335.1 | *Arthrobacter creatinolyticus* strain E3.As6 |
| KJ513334.1 | *Lysinibacillus* sp strain E3.As5 |
| KJ513333.1 | *Lysinibacillus boronitolerans* strain E3.As4 |
| KJ513332.1 | *Arthrobacter creatinolyticus* strain E3.As3 |
| KJ513331.1 | *Bacillus subtilis* group strain E3.As2 |
| KJ513330.1 | *Acinetobacter radioresistens* strain E3.As1 |
| KJ513329.1 | *Actinomyces oris* strain E3.Pb7 |
| KJ513328.1 | *Actinomyces johnsonii* strain E3.Pb6 |
| KJ513327.1 | *Actinomyces viscosu*s strain E3.Pb5 |
| KJ513326.1 | *Enterobacter cloacae* complex strain E3.Pb4 |
| KJ513325.1 | *Lysinibacillus contaminans* strain E3.Pb3 |
| KJ513324.1 | *Enterococcus casseliflavus* strain E3.Pb2 |
| KJ513323.1 | *Bhargavaea sp.* strain E3.Pb1 |
| KJ513322.1 | *Bacillus cereus* group strain E2.Zn2 |
| KJ513321.1 | *Bacillus cereus* group strain E2.Zn1 |
| KJ513320.1 | *Bacillus cereus* group strain E2.Cu2 |
| KJ513319.1 | *Bacillus cereus* group strain E2.Cu1 |
| KJ513318.1 | *Enterococcus faecalis* strain E2.Cd4 |
| KJ513317.1 | *Enterococcus casseliflavus* strain E2.Cd3 |
| KJ513316.1 | *Bacillus cereus* group strain E2.Cd2 |
| KJ513315.1 | *Tessaracoccus profundi* strain E2.Cd1 |
| KJ513314.1 | *Lysinibacillus contaminans* strain E2.As6 |
| KJ513313.1 | *Bacillus cereus* group strain E2.As5 |
| KJ513312.1 | *Lysinibacillus* sp. strain E2.As4 |
| KJ513311.1 | *Bacillus cereus* group strain E2.As3 |
| KJ513310.1 | *Dietzia cinnamea* strain E2.As2 |
| KJ513309.1 | *Lysinibacillus sphaericus* strain E2.Pb4 |
| KJ513308.1 | *Enterobacter asburiae* strain E2.Pb3 |
| KJ513307.1 | *Paenibacillus apiariu*s strain E2.Pb2 |
| KJ513306.1 | *Paenibacillu*s sp. strain E2.Pb1 |
| KJ513305.1 | *Bacillus cereus* group strain E1.Zn1 |
| KJ513304.1 | *Bacillus cereus* group strain E1.Pb1 |
|  |  |

**Supplementary Figure S1 online:** Five different collection sites: process water, pumped from the depurated border of the tailing pond, in which the ore-processing wastes are discharged (E1); floater surfaces, in which chalcopyrite is separated from the other ores, and which uses both amyl xanthate and xanthate ester as collectors, and methyl isobutyl carbinol and glycol propylene as frothers (E2); the discharging point, where the ore processing wastes are thrown in the tailing pond (E3); the edge of the tailing pond, where the waste particles are sedimented (E4); the soil close to the edge of the tailing pond, which is affected by the deposited waste (E5).


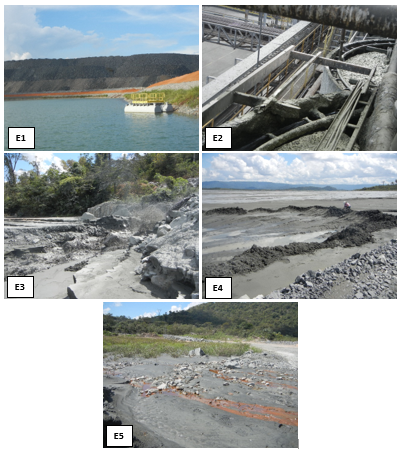

Supplement: Supplementary file 1 — Supplementary info. [file 41598_2020_62780_MOESM1_ESM.docx]
